# Femoral vein thrombophlebitis and septic pulmonary embolism due to a mixed anaerobic infection including *Solobacterium moorei*: a case report

**DOI:** 10.1186/1752-1947-1-40

**Published:** 2007-07-02

**Authors:** Claire A Martin, Rohan S Wijesurendra, Colin DR Borland, Johannis A Karas

**Affiliations:** 1 Department of Medicine, Hinchingbrooke Hospital, Hinchingbrooke Heath Care NHS Trust, Huntingdon, Cambridgeshire, PE29 6NT, UK; 2 Department of Microbiology, Hinchingbrooke Hospital, Hinchingbrooke Heath Care NHS Trust, Huntingdon, Cambridgeshire, PE29 6NT, UK

## Abstract

**Background:**

Primary foci of necrobacillosis infection outside the head and neck are uncommon but have been reported in the urogenital or gastrointestinal tracts. Reports of infection with *Solobacterium moorei *are rare.

**Case presentation:**

A 37-year-old male intravenous drug user was admitted with pain in his right groin, fever, rigors and vomiting following a recent injection into the right femoral vein. Admission blood cultures grew *Fusobacterium nucleatum, Solobacterium moorei *and *Bacteroides ureolyticus*. The patient was successfully treated with intravenous penicillin and metronidazole.

**Conclusion:**

This case report describes an unusual case of femoral thrombophlebitis with septic pulmonary embolism associated with anaerobic organisms in a groin abscess. *Solobacterium moorei*, though rarely described, may also have clinically significant pathogenic potential.

## Background

*Fusobacterium nucleatum *is a strictly anaerobic Gram-negative bacillus. It is generally considered to be a commensal of the human oropharynx but is also documented to cause severe infections including necrobacillosis [1]. In order to promote an anaerobic environment suitable for their growth, Fusobacterium species aggregate human platelets and promote intravascular coagulation. The thrombo-embolic phenomena that result account for much of the morbidity associated with necrobacillosis.

*Bacteroides spp *are a heterogeneous group of Gram-negative obligate anaerobes. They are common gut commensals but also opportunistic pathogens, mostly causing intra-abdominal abscesses in cases where the mucosal wall of the intestine is disrupted. They are also part of the oral flora and can cause peri-oral infection. Bacteroides contribute to development of a synergistic infection by reducing phagocytosis by polymorphs and through inactivation of antibiotics by β-lactamase production.

*Solobacterium spp *are anaerobic Gram-positive bacteria known to exist in the oropharynx, and probably involved in causing halitosis. Reports of disseminated infection caused by Solobacterium spp are very rare, with a recent paper claiming the first recovery of Solobacterium moorei from blood cultures in a septic patient with multiple myeloma [2]. A further report gives a case of bacteraemia caused by Solobacterium moorei in a patient with acute proctitis and carcinoma of the cervix [3].

The most common presentation of necrobacillosis is as Lemierre's syndrome usually caused by *Fusobacterium necrophorum *but other organisms have also been implicated [4,5]. This is characterised by pharyngotonsillitis, internal jugular vein thrombophlebitis and septic embolisation most commonly affecting the lungs. Primary foci of necrobacillosis infection outside the head and neck are uncommon but have been reported in the urogenital or gastrointestinal tracts. We present a case of femoral thrombophlebitis and septic pulmonary embolism due to a mixed anaerobic infection including *Solobacterium moorei*.

## Case presentation

A 37-year-old male intravenous drug user was admitted feeling generally unwell with pain in his right groin. Following a recent injection into the right femoral vein, his right groin had become more red, swollen and painful followed by systemic symptoms of fever, rigors and vomiting. His only past medical history was of a left groin deep venous thrombosis 2 years previously and he was taking no regular medications.

His temperature was 39.4°C, blood pressure 129/62 mmHg and heart rate 110 beats min^-1^. Physical examination showed multiple injection sites and an erythematous right groin, with bilateral groin sinuses and some lymphadenopathy on palpation. Cardiovascular, respiratory and abdominal examination was unremarkable.

Analysis of blood showed haemoglobin 8.4 gdl^-1^, white cell count 12.3 × 10^9 ^L^-1^, absolute neutrophils 9.6 × 10^9 ^L^-1^, C-reactive protein 345 mg L^-1^. Urinalysis and chest radiograph were normal and electrocardiogram revealed a sinus tachycardia. Three sets of blood cultures were taken, one from a dorsal foot vein and two sets from the left radial artery.

Treatment was initiated with intravenous benzyl penicillin 1.2 g six-hourly and flucloxacillin 2 g six-hourly and subcutaneous low molecular weight heparin.

A trans-thoracic echocardiogram showed an echogenic lesion in the inferior vena cava associated with the Eustachian valve and heart valves free of vegetations. An ultrasound examination of the groin showed a completely thrombosed right superficial femoral vein, and a 1 × 1.5 cm echogenic area that was consistent with either a lymph node or an abscess.

The patient's condition failed to improve and he continued to spike temperatures of up to 40°C several times per day. He became progressively more unwell with hypotension, lactic acidosis, thrombocytopenia and anaemia. On day 6 of his admission, the patient began to feel more short of breath and complained of pleuritic chest pain, and he was noted to be hypoxic with generalised wheeze and a right-sided pleural rub on examination. A repeat trans-thoracic echocardiogram showed no progression of the lesion in his inferior vena cava. A computed tomography examination revealed numerous small opacities in both lungs, some of which had low attenuating centres and appeared to represent small abscesses [see figure]. One anaerobic blood culture (BacT/Alert 3D BioMérieux) bottle taken at admission had by this time become positive. This revealed Gram-negative anaerobic rods morphologically resembling Fusobacterium and intravenous clindamycin 400 mg six-hourly started. Subsequently two further anaerobic blood culture bottles became positive. The organisms were identified as *Fusobacterium nucleatum*, *Bacteroides ureolyticus *and *Solobacterium moorei *by the national anaerobic reference laboratory (PHLS Wales, Cardiff). The method of identification used was the 16S rDNA restriction analysis as previously described [6,7]. A diagnosis of septic pulmonary embolism was made and the anti-microbial therapy was changed to intravenous metronidazole 500 mg eight-hourly and benzylpenicillin 1.2 g six-hourly.

The patient became apyrexial and his clinical condition and inflammatory markers improved dramatically – by day 17 of admission his C-reactive protein had decreased to 5 mg L^-1^. He was discharged on oral antibiotics and subcutaneous low molecular weight heparin to continue in the community.

Our patient's likely source of infection was the abscess in the right superficial femoral vein, at the site of previous intravenous injection. It is possible that his own oral flora were inoculated in the soft tissue abscess in his leg. This abscess probably induced inferior vena cava thromboses and septic pulmonary emboli. Septic embolism in necrobacillosis most commonly results in pleuro-pulmonary infections with brain and liver abscesses, meningitis, septic arthritis, osteomyelitis, and endocarditis also described. This case is unusual as metastatic embolisation is rare in patients with foci of infection outside the head and we only found two other cases in the literature both due to *F. necrophorum *and not *F. nucleatum *as in this case – one complicated by portal vein thrombosis [8] and another case of soft tissue abscess complicated by inferior vena cava thrombosis [9].

There is limited evidence for the use of anticoagulant therapy for necrobacillosis-associated thrombosis. Whilst there is a theoretical risk of promoting the spread of infection, gynaecological studies have shown benefit in anticoagulation for pelvic septic thrombophlebitis, especially in patients with clot propagation despite antimicrobial therapy [10]. We anti-coagulated the patient in view of his large and propagating superficial femoral vein thrombosis.

## Conclusion

Our case emphasizes the local thrombogenic potential of necrobacillosis organisms, with extensive superficial femoral vein thromboses in proximity to the groin abscess and the ability to cause septic embolisation with seeding to the inferior vena cava and to the lungs. We advocate the need for a high degree of clinical suspicion, an early diagnosis, and prompt institution of effective antimicrobial therapy to decrease the mortality and morbidity associated with septic pulmonary embolisation.

To our knowledge, this is the first report of superficial femoral vein thrombosis with pulmonary and inferior vena cava emboli associated with anaerobic organisms in a groin abscess. *Solobacterium moorei*, though rarely described, may also have clinically significant pathogenic potential.

## Competing interests

The author(s) declare that they have no competing interests.

## Authors' contributions

CM, RW, CB for clinical and AK for laboratory work, all contributed to the writing of the article. All authors have seen and approved the final manuscript.

**Figure 1:**
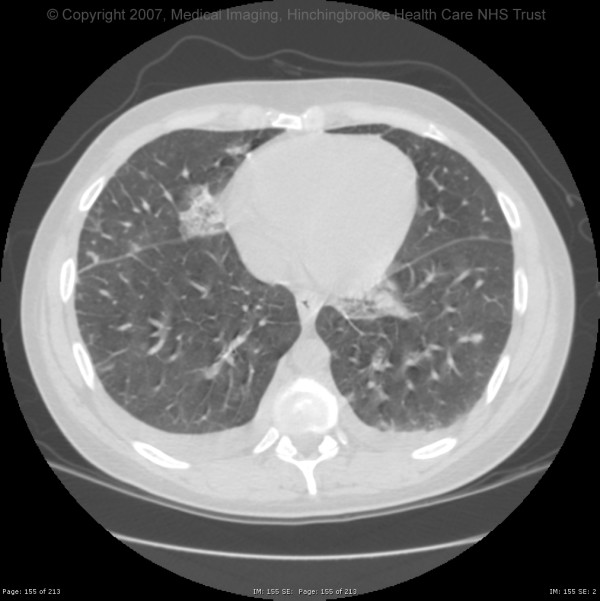
Computed tomography of the chest showing multiple cavitating lung lesions.
